# Metropolitan Hospital Sunday Fund

**Published:** 1899-07-29

**Authors:** 


### METROPOLITAN HOSPITAL SUNDAY FUND.

### PARTICULARS OF AWARDS.

THE following is the report of the Committee of Distribution adopted by the Council of the Metropolitan Hospital Sunday Fund on Wednesday (26th inst.): The Committee of Distribution recommend awards this year to the 192 institutions shown in the appendix attached hereto (given below), being four more than last year. The amount collected this year, including ¾ interest from the Court of Chancery, amounts to £50,716, and the committee anticipate a further £2,000 before the accounts are closed on October 31st. The distribution of £50,500 to the 138 hospitals, &c., and to the 54 dispensaries, is now recommended.

This year 195 institutions have made applications for grants from the funds at your disposal. Of this number it was found necessary to invite the attendance of deputations from the governing bodies of eighteen. Your committee are not able to recommend any awards in three cases. Deputations from twelve hospitals and from two dispensaries conferred with your committee. Explanations modifying the causes of adverse criticism were given in several cases and the awards are as now recommended.

In addition to the £50,500 recommended for distribution, 5 per cent. of the total sum collected—viz., about £2,500—is set apart to purchase surgical appliances (in obedience to Law XII.), in monthly proportions during the ensuing year.

In compliance with an order of the Council, and for the special use of its members, tables of statistics have been prepared as usual, showing an analysis of the number of beds in hospitals, the cost of patients both at hospitals and dispensaries, the proportionate expense of management, as well as other valuable information, and copies are sent to every member of the Council.

The amount available for distribution is larger than that distributed last year, owing to the munificent donation of £10,000 from Mr. George Herring. The collections on Hospital Sunday are generally larger than those of last year, and show that the confidence of the public in the Metropolitan Hospital Sunday Fund is well sustained.

THIRTY-ONE GENERAL HOSPITALS.

Charing Cross Hospital, £1,270 16s. 8d.; French Hospital, £354 3s. 4d.; German Hospital, £625; Great Northern Central Hospital, £625; Guy's Hospital, £1,562 10s.; Hampstead Hospital, £208 6s. 8d.; Italian Hospital, £62 10s.; King's College Hospital, £1,664 13s. 4d.; London Hospital, £5,000; London Homœopathic Hospital, £625; Phillips Memorial Homœopathic Hospital, £36 9s. 2d.; London Temperance Hospital, £802 1s. 8d.; Metropolitan Hospital, £729 3s. 4d.; Mildmay Mission Hospital, £312 10s.; Miller Hospital and Royal Kent Dispensary, £286 9s. 2d.; North-West London Hospital, £416 13s. 4d.; Poplar Hospital, £729 3s. 4d.; Queen's Jubilee Hospital, nil; Royal Free Hospital, £1,093 15s.; St. George's Hospital, £2,083 6s. 8d.; SS. John and Elizabeth Hospital, £78 2s. 6d.; St. Mary's Hospital, £1,927 1s. 8d.; St. Thomas's Hospital, £104 3s. 4d.; Seamen's Hospital Society, £1,145 16s. 8d.; the Middlesex Hospital, £2,187 10s.; Tottenham Hospital, £260 8s. 4d.; University College Hospital, £1,406 5s.; Walthamstow, £88 10s. 10d.; West Ham Hospital, £406 5s.; West London Hospital, £625; Westminster Hospital, £1,458 6s. 8d.

SPECIAL HOSPITALS.

FIVE CHEST HOSPITALS.—City of London Hospital for Diseases of the Chest, Victoria Park, £937 10s.; Hospital for Consumption, Brompton, £1,822 18s. 4d.; North London Consumption Hospital, Hampstead, £364 11s. 8d.; Royal Hospital for Diseases of the Chest, City Road, £416 13s. 4d.; Royal National Hospital for Consumption (Ventnor), £260 8s. 4d.

SEVENTEEN CHILDREN'S HOSPITALS.—Alexandra Hospital for Hip Disease, W.C., £171 17s. 6d.; Banstead Surgical Home, £41 13s. 4d.; Barnet Home Hospital, £52 1s. 8d.; Belgrave Hospital for Children, S.W., £135 8s. 4d.; Cheyne Hospital for Incurable Children, S.W., £125; East London Hospital for Children, Shadwell, E., £677 1s. 8d.; Evelina Hospital for Sick Children, Southwark, S.E., £468 15s.; Home for Incurable Children, Maida Vale, W., £78 2s. 6d.; Home for Sick Children, Sydenham, S.E., £135 8s. 4d.; Hospital for Sick Children, Great Ormond Street, W.C., £781 5s.; North-Eastern Hospital for Children, Hackney Road, N.E., £364 11s. 8d.; Paddington Green Hospital for Children, W., £260 8s. 4d.; St. Mary's, Plaistow, E., £171 17s. 6d.; St. Monica's, Brondesbury, N.W., £104 3s. 4d.; Victoria Hospital for Children, King's Road, Chelsea, S.W., £625; Victoria Home, Margate, £31 5s.; The Vine, Sevenoaks, £41 13s. 4d.

FIVE LYING-IN HOSPITALS.—British Lying-in Hospital, Endell Street, W.C., £36 9s. 2d.; Clapham Maternity Hospital, £36 9s. 2d.; East End Mothers' Home, £88 10s. 10d.; General Lying-in Hospital, Lambeth, S.E., £52 1s. 8d.; Queen Charlotte's Lying-in Hospital, Marylebone Road, W., £312 10s.

SIX HOSPITALS FOR WOMEN.—Chelsea Hospital for Women, S.W., £364 11s. 8d.; Hospital for Women, Soho Square, W., £312 10s.; Grosvenor Hospital for Women and Children, Vincent Square, S.W., £83 6s. 8d.; New Hospital for Women, Euston Road, W.C., £260 8s. 4d.; Royal Hospital for Children and Women, Lambeth, S.E., £229 3s. 4d.; Samaritan Free Hospital, Lower Seymour Street, W., £416 13s. 4d.

TWENTY-SIX OTHER SPECIAL HOSPITALS.

Cancer Hospital, Brompton, S.W., £625; London Fever Hospital, Islington, N., £1,041 13s. 4d.; Gordon Hospital for Fistula, Vauxhall Bridge Road, S.W., £38 10s. 10d.; St. Mark's Hospital for Fistula, City Road, E.C., £156 5s.; National Hospital for the Diseases of the Heart and Paralysis, Soho Square, W., £83 6s. 8d.; Female Lock Hospital, Harrow Road, W., £197 18s. 4d.; Hospital for Epilepsy, Paralysis, and other Diseases of the Nervous System, Regent's Park, N.W., £67 14s. 2d.; National Hospital for the Paralyzed and Epileptic, W.C., £781 5s.; West End Hospital for Diseases of the Nervous System, £125; Central London Ophthalmic Hospital, Gray's Inn Road, W.C., £46 17s. 6d.; Royal Eye Hospital, St. George's Circus, S.E., £208 6s. 8d.; Royal London Ophthalmic Hospital, Moorfields, E.C., £625; Royal Westminster Ophthalmic Hospital, Charing Cross, W.C., £125; Western Ophthalmic Hospital, Marylebone Road, W.; £41 13s. 4d.; City Orthopædic Hospital, Hatton Garden, E.C., £83 6s. 8d.; National Orthopædic Hospital, Great Portland Street, W., £104 3s. 4d.; Royal Orthopædic Hospital, Oxford Street, W., £78 2s. 6d.; Royal Sea-Bathing Hospital, Margate, £520 16s. 8d.; Hospital for Diseases of the Skin, Stamford Street, S.E., £10 8s. 4d.; St. Peter's Hospital for Stone, Covent Garden, £41 13s. 4d.; Central London Throat and Ear Hospital, Gray's Inn Road, W.C., £41 13s. 4d.; London Throat Hospital, Great Portland Street, W., £31 5s.; Metropolitan Ear and Nose Hospital, nil; Royal Ear Hospital, Frith Street, W., £10 8s. 4d.; Dental Hospital of London, Leicester Square, W.C., £130 4s. 2d.; National Dental Hospital, 149, Great Portland Street, W., £83 6s. 8d.

TWENTY-NINE CONVALESCENT HOSPITALS.

Metropolitan Convalescent Institution, Sackville Street, W., £625; Bexhill Convalescent Home, Sackville Street, W., £343 15s.; All Saints'

Convalescent Hospital, Eastbourne, £416 13s. 4d.; Ascot Priory Convalescent Home, £57 5s. 10d.; Chelsea Hospital for Women Convalescent Home, St. Leonards, £41 13s. 4d.; Mrs. Gladstone's Convalescent Home, Woodford, £62 10s.; Hahnemann Convalescent Home, Bournemouth, £31 5s.; Hanwell Convalescent Home, £27 1s. 8d.; Herbert Convalescent Home, Bournemouth, £20 16s. 8d.; Herne Bay Baldwin-Brown Convalescent Home, £36 9s. 2d.; Herne Bay Mothers' Home, £43 15s.; Homœopathic Convalescent Home, Eastbourne, £20 16s. 8d.; King's College Hospital Convalescent Home, Hemel Hempstead, £62 10s.; Mrs. Kitto's Convalescent Home, Reigate, £83 6s. 8d.; London and Brighton Female Convalescent Home, £10 8s. 4d.; Mary Wardell Convalescent Home for Scarlet Fever, £104 3s. 4d.; Morley House Convalescent Home for Working Men, £156 5s.; Police Seaside Home, Brighton, £52 1s. 8d.; St. Andrew's Convalescent Hospital, Clewer, £156 5s.; St. Andrew's Convalescent Home, Folkestone, £281 5s.; St. John's Home for Convalescent and Crippled Children, Brighton, £114 11s. 8d.; St. Joseph's Convalescent Home, Bournemouth, £52 1s. 8d.; St. Leonard's-on-Sea Convalescent Home for Poor Children, £104 3s. 4d.; St. Mary's Convalescent Home, Shortlands, £16 13s. 4d.; St. Michael's Convalescent Home, Westgate-on-Sea, £31 5s.; Seaside Convalescent Hospital, Seaford, £145 16s. 8d.; Warley Convalescent Cottage Hospital, Essex, £10 8s. 4d.; Friendly Societies' Convalescent Home, Dover, £20 16s. 8d.; Deptford Medical Mission Convalescent Home, Bexhill, £20 16s. 8d.

Fourteen Cottage Hospitals.

Beckenham Cottage Hospital, £52 1s. 8d.; Blackheath and Charlton Cottage Hospital, £72 18s. 4d.; Bromley, Kent, Cottage Hospital, £88 10s. 10d.; Chislehurst, Sidcup, and Cray Valley Cottage Hospital, £41 13s. 4d.; Eltham Cottage Hospital, £31 5s.; Enfield Cottage Hospital, £50; Epsom and Ewell Cottage Hospital, £57 5s. 10d.; Hounslow Cottage Hospital, £18 15s.; Livingstone Cottage Hospital, £52 1s. 8d.; Mildmay Cottage Hospital, £62 10s.; Reigate and Redhill Cottage Hospital, £67 14s. 2d.; Sidcup Cottage Hospital, £38 10s. 10d.; Tilbury (Passmore Edwards) Cottage Hospital, £26 0s. 10d.; Wimbledon Cottage Hospital, £31 5s.

Seven Institutions.

Establishment for Gentlewomen, Harley Street, £133 6s. 8d.; St. Saviour's Hospital and Nursing Home, £122 18s. 4d.; National Sanatorium for Consumption, Bournemouth, £83 6s. 8d.; Invalid Asylum, Stoke Newington, £62 10s.; Firs Home, Bournemouth, £15 12s. 6d.; St. Catherine's Home, Ventnor, £31 5s.; Friedenheim Home for the Dying, £260 8s. 4d.

Fifty-five Dispensaries.

Battersea Provident Dispensary, £88 10s. 10d.; Bloomsbury Provident Dispensary, £11 9s. 2d.; Brixton, &c., Dispensary, £52 1s. 8d.; Brompton Provident Dispensary, £25; Camberwell Provident Dispensary, £88 10s. 10d.; Camden Provident Dispensary, £12 10s.; Chelsea, Brompton, and Belgrave Dispensary, £45 16s. 8d.; Chelsea Provident Dispensary, £15 12s. 6d.; Child's Hill Provident Dispensary, £11 9s. 2d.; City Dispensary, £114 11s. 8d.; City of London and East London Dispensary, £26 0s. 10d.; Clapham General and Provident Dispensary, £27 1s. 8d.; Deptford Medical Mission, £20 16s. 8d.; Eastern Dispensary, £31 5s.; East Dulwich Provident Dispensary, £29 3s. 4d.; Farringdon General Dispensary, £39 11s. 8d.; Finsbury Dispensary, £15 12s. 6d.; Forest Hill Dispensary, £41 13s. 4d.; Hackney Provident Dispensary, £11 9s. 10d.; Hampstead Provident Dispensary, £41 13s. 4d.; Holloway and North Islington Dispensary, £57 5s. 10d.; Islington Dispensary, £46 17s. 6d.; Kensal Town Provident Dispensary, £10 8s. 4d.; Kensington Dispensary, £67 14s. 2d.; Kilburn, Maida Vale, and St. John's Wood Dispensary, £41 13s. 4d.; Kilburn Provident Medical Institution, £36 9s. 2d.; London Dispensary, £20 16s. 8d.; London Medical Mission, Endell Street, £93 15s.; Margaret Street Infirmary for Consumption, nil; Metropolitan Dispensary, £57 5s. 10d.; Notting Hill Dispensary and Maternity (Provident), £10 8s. 4d.; Paddington Provident Dispensary, £34 7s. 6d.; Pimlico Provident Dispensary (Lupus Street), £18 15s.; Portland Town Dispensary, £17 14s. 2d.; Public Dispensary, £41 13s. 4d.; Queen Adelaide's Dispensary, £18 15s.; Royal General Dispensary, £36 9s. 2d.; Royal Pimlico Provident Dispensary, £67 14s. 2d.; Royal South London Dispensary, £46 17s. 6d.; St. George's and St. James's Dispensary, £46 17s. 6d.; St. George's, Hanover Square, Provident Dispensary, £36 9s. 2d.; St. John's Wood and Portland Town Provident Dispensary, £35 8s. 4d.; St. Marylebone General Dispensary, £45 16s. 8d.; St. Pancras and Northern Dispensary, £37 10s.; South Lambeth, Stockwell, and North Brixton Dispensary, £46 17s. 6d.; Stamford Hill, Stoke Newington, &c., Dispensary, £50; Tower Hamlets Dispensary, £41 13s. 4d.; Walworth Provident Dispensary, £10 8s. 4d.; Wandsworth Common Provident Dispensary, £8 6s. 8d.; Westbourne Provident Dispensary and Maternity, £13 10s. 10d.; Western Dispensary, £54 3s. 4d.; Western General Dispensary, £135 8s. 4d.; Westminster General Dispensary, £31 5s.; Whitechapel Provident Dispensary, £20 16s. 8d.; Woolwich Provident Dispensary, £11 9s. 2d.

SEAMEN'S HOSPITAL SOCIETY,

